# Morbidity profile in a rural community-based rehabilitation programme in Butembo, North Kivu Province, Congo

**DOI:** 10.4102/phcfm.v3i1.215

**Published:** 2011-06-17

**Authors:** Prosperine V. Masika, Prosper M. Lutala

**Affiliations:** 1 Faculty of Medicine, Catholic University of Graben, Congo (DRC); 2 Faculty of Medicine, University of Goma, Congo (DRC)

## Abstract

**Background:**

Medicine in low socio-economic countries is primarily disease-oriented; prevention and rehabilitative care are secondary concerns. Hence, curative care erodes the few resources allocated to health. Despite the well-documented benefits of community-rehabilitation in the management of chronic conditions, little is known about common conditions present in the community in Butembo.

**Objectives:**

The objective of this study was to determine the conditions encountered during rehabilitation in Butembo and to identify the trends of the five most common conditions during the study period.

**Method:**

Data were extracted from a rehabilitation programme connect to one centre in Butembo. A descriptive retrospective medical study was performed for the period between 2004 and 2007. Descriptive statistics with percentages were computed. The Chi-square test was used to evaluate the differences with a probability of 5%.

**Results:**

Cerebral palsy (46.9%), cataract (17.3%), clubfoot (11.8%), glaucoma (6.8%), and cleft lip (4.5%) were the most commonly encountered conditions, with cerebral palsy the most common condition throughout the study period. With regard to gender, male patients were significantly more affected by cataracts (*p* = 0.0290), clubfoot (*p* < 0.0100) and glaucoma (*p* < 0.0100) than female patients. Children aged five or younger had a higher incidence of cerebral palsy (*χ^2^* = 263.2, df = 1, *p* = 0.0000) cataract (*p* = 0.0170), clubfoot (*p* < 0.0010), and glaucoma (*p* = 0.0010). Additionally, the overall comparisons by gender and age demonstrated differences for the five most common conditions (*χ^2^* = 15.3, df = 4, and *p* = 0.0040; and *χ^2^* = 114, df = 4, and *p* < 0.0001 for gender and age, respectively).

**Conclusion:**

Common conditions and associated factors were identified that will add to the effectiveness of the programme in terms of materials needed, staff skills, and programming. Special skills are still needed to help treat some acute conditions that can be handled at the rehabilitation centre, and a triage of attending rehabilitation centres could improve the effectiveness of the programme and lower the possibility of missed opportunities for acute stage patients.

## Introduction

### Setting

#### Key focus

This study will be in aid for planning a rehabilitation service in Butembo and similar settings in the allocation of resources, the deployment of staff and in the composition of the kits.

#### Background

Medical interventions in many developing countries, including Congo, are completely cure-oriented. Patients as well as providers are mostly oriented towards the treatment of diseases rather than the prevention of diseases or the rehabilitation of so-called ‘chronic disability diseases’. It follows that all efforts are focused on expensive medication and investigations, and little resources are allocated for the prevention of disease and the improvement of quality of life. As a result the health system is struggling to treat conditions that do not respond to curative measures, eroding the already scarce allocated resources. It was felt that without rehabilitation, people with disabilities and chronic illnesses may be forced to live in institutional settings to access health care, and those who stay in the community may either have their lives at risk or be highly dependent on family members.^1^

The role that patients’ families play in rehabilitation programmes cannot be overemphasised. Whilst providing care in the community or family context, community rehabilitation allows injured people, for example, to remain part of the family and community life, participate in normal activities and responsibilities, and become an active and contributing member of his or her social groups.^2^ Hence, patients can have family members with whom they share joys and achievements, decision making and assistance when required, all of which leads to more satisfaction.^3^

The family also increases the assistance provided to older persons in rehabilitation centers^4^ and helps with carrier decisions^5^ once the patient is socially integrated. To provide help in performing everyday activities, to facilitate interactions with people with disabilities or sick people, and to provide assistance in treatment prior to any contact with a point of care,^6^ are additional roles fulfilled by families in rehabilitation programmes. Finally, family caregivers play a role in the medication adherence of their family members.^7^ However, from the caregiver's perspective, the role of family members in rehabilitation is not usually positive. The role of family members was, for example, perceived as restrictive for patients in rehabilitation programmes for stroke^8^ when they were discouraging patients from travelling outdoors for fear of recurrence, falling, losing their way, or an inability to manage the distance.

#### Trends

Several attempts to redress the situation have been undertaken with little success. With the spread of several non-government organisations, a focus on rehabilitation in the community using different approaches was suggested, one of them being a community-based approach (CBR). CBR is a strategy for rehabilitation, equalisation of opportunities, and social inclusion of all children and adults with disabilities.^9^

This approach has shown several advantages such as high effectiveness and value for people with disabilities in the community^10, 11^ by making it easier to integrate people with disabilities through education programmes^12^ and by making it possible to train generic community workers in the delivery of rehabilitation and prevention services to people with disabilities and their families.^13, 14^ Congenital malformations account for a large proportion of chronic disabilities.^15^ The morbidity and disability experienced by surviving children with disabilities, who comprise a large portion of rehabilitation services users, also have a major public health impact.^16^

The management itself of such clients is time-consuming, and requires sufficient resources for ‘few direct results’ in the end. All of this requires good planning in terms of staff, equipment, time, resources, and even prior knowledge of common conditions encountered in the programme.

#### Objectives

We decided therefore firstly, to identify conditions encountered by rehabilitative teams in the Butembo zone and surrounding areas and to correlate them to the gender and age of the participants. Secondly, we sought to identify the trends of the five most common conditions during the study period.

#### Contribution to field

This study describes conditions encountered by the centre, which will serve in planning rehabilitation services in Butembo and similar settings regarding the allocation of resources, deployment of staff, and the composition of the kits.

## Ethical considerations

### Potential benefits and hazards

There were no direct potential physical, psychological, or disclosure danger that could be anticipated for the study. However, similar to several other studies, this study provides benefits for the participants and for society in general by providing data currently unavailable on common conditions of patients consulting rehabilitation services that could be used to improve service delivery. The minor risk of discrimination that could arise from the disclosure of participants was minimised by removing all participant identifiers and keeping them locked in a file with a password. We obtained approval to conduct the study from the rehabilitation service. No additional measure was necessary to protect data, as the data were collected from a place where all universal data protections regulations were already applied in the database. Informed consent was not sought for this paper for several reasons: its retrospective nature with no blanket informed consent; furthermore, this paper does not report on primary data. In addition, patients have been treated in line with the national guidelines, and the paper does not report on experimental data or new guidelines. It should also be kept in mind that this programme was not designed as a research project or a new protocol but as a treatment programme, and finally that the study was carried out as an audit or evaluation of the programme of rehabilitation.

## Methods

### Materials

The population from which the sample was taken included all clients or patients attended to on site or receiving outreach at a rehabilitation centre called ‘Heshima Letu’ in Butembo.

Successive participants attending the centre were included in the study irrespective of the nature and acuity of their conditions. Some participants travelled from their homes to consult a rehabilitation team directly at the centre, but most participants visited outreach sites during community visits. Patients of all ages and both sexes were included.

### Setting

Our study was performed in Butembo, Province of Nord-Kivu in the Eastern Congo. It is a city inhabited by 700 000 people subdivided in four parts. Butembo is 19.034 km^2^ in size and is located at an altitude between 1630 m to 2000 m. Its latitudinal and longitudinal location is between 29° and 29°30′ E and 0° and 0°16′ N, respectively. Heshima Letu is the centre where the study was conducted.

This programme is an outreach programme that started as an orthopaedics programme before expanding its activities to common chronic conditions. It was not intended as a substitute or an alternative to mainstream medical services, and government officials continued to be in charge of the hospitalisation of severe cases, the treatment of patients with complications or acute episodes, and the referral of some of the clients who were not in the project.

The team was composed of medical doctors (general practitioners and surgeons), community nurses, physiotherapists, patient attendants, and community volunteers. Patients or clients were coming for assessment, follow-up, referral, massage or treatment, and those with operable or severe conditions were referred to the district hospital for further investigation and management.

### Design

This study was a descriptive retrospective study of a rehabilitation centre.

### Procedure

After obtaining approval from the management team, registers of patients or clients who consulted between 2004 and 2009 were reviewed by the first-mentioned author. Data were collected after checking for completeness of data regarding variables of interest in this study, including age, gender, period of consultation, and type of abnormality. All recorded patients with incomplete data in the registers were excluded from the study (Table 2).

### Analysing

All compiled data were screened for completeness and accuracy. A reviewed diagnosis were made during the visits made by a multidisciplinary team composed of an orthopaedic surgeon, an eye specialist, community nurses, a rehabilitation technician. This was recorded in the data collection spreadsheet for cases that were selected using a simple random sampling method. Thereafter, data were transferred to the Statistical Package for Social Science (SPSS version 16) for descriptive statistical analysis. Percentages were calculated, and a graph of the trends of common diseases was plotted for the period covered by the study. Descriptive statistics were computed. Chi-square tests were used for categorical data to compare the five most common conditions regarding age and gender. The one-way Chi-squared test with Yates correction for continuity (that is, df = 1) was used to verify whether a frequency distribution fits a specific pattern for each of the five common conditions by gender and age range. Significance was set at *p* < 0.0500.

## Results

In total, 638 participants were enrolled in the study. Amongst these participants, 375 (58.8%) were male, and 263 (41.2%) were female. The distribution of the ages of male participants were as follow, 324 (86.4%) participants were aged five or younger, whilst 51 (13.6%) were between 6 and 18 years old. In regard to female participants, 228 (86.6%) were under 5 years old, whilst 35 (13.3%) were between 6 and 18 years old. The mean age of participants was 10 years (with the standard deviation [s.d.] approximately 2 years). Cataracts, retinoblastoma, glaucoma, and blindness were the most common eyes conditions; whilst amongst orthopaedic conditions, cerebral palsy, hydrocephalus, and clubfoot were predominant (Table 1). Most participants were less than 5 years old (552/638, 86.5% of the sample).

**TABLE 1:** Morbidity by gender and age ranges of participants.

Gender conditions	Male participants (*n* = 375)					Female participants (*n* = 263)				Total
		
	Age (years)		*n*	%		Age (years)		*n*	%	
		
	≤ 5	5–18				≤ 5	5–18			
Cerebral palsy	158	1	159	53		133	8	141	47	300
Cataract	35	32	67	60.3		33	11	44	39.6	111
Clubfoot	51	3	54	71		21	1	22	28.9	76
Glaucoma	28	6	34	77.2		9	1	10	22.7	44
Cleft lip or palate	12	6	18	62		5	6	11	37.9	29
Retinoblastoma	11	0	11	47.8		12	0	12	52.1	23
Blindness	9	0	9	47.3		7	3	10	52.6	19
Hydrocephalus	9	0	9	64.2		5	0	5	35.7	14
Spina bifida	6	0	6	85.7		1	0	1	14.2	7
Hypospadias	1	1	2	50		0	2	2	50	4
Polydactyly	2	0	2	66.6		0	1	1	33.3	3
Syndactyly	1	2	3	100		0	0	-	0	3
Chorioretinitis	0	0	-	0		1	1	2	100	2
Microphtalmia	1	0	1	50		0	1	1	50	2
Imperforated anus	0	0	-	0		1	0	1	100	1

*Source*: Authors’ original data

*n*, given as means of number.

There were statistically significant differences amongst the five groups of common conditions (Table 2) regarding gender (*χ*^2^ = 15.300, *df* = 4, *p* < 0.0040) and age (*χ*^2^ = 114.000, *df* = 4, and *p* = 0.0000). With regard to gender, a statistical difference between male participants and female participants was found for incidence of cataract (*χ*^2^ = 4.360, *df* = 1, *p* = 0.0290), clubfoot (*χ*^2^ = 12.645; *df* = 1, *p* < 0.0100) and glaucoma (*χ*^2^ = 12.030; *df* = 1, *p* < 0.0100). A statistical difference was found between participants aged less than 5 years and those aged 6*–*14 years, for the incidence of cerebral palsy (*χ*^2^ = 263.200, *df* = 1, *p* < 0.0001), for cataracts(*χ*^2^ = 2.860; *df* = 1, *p* = 0.0017), for clubfoot (*χ*^2^ = 59.660; *df* = 1, *p* < 0.0010), and for glaucoma (*χ*^2^ = 11.600; *df* = 4, *p* = 0.0010).

**TABLE 2a:** Variance of different morbidities by gender.

Gender	Male participants (*n* = 332)		Female participants (*n* = 228)		Total	*χ*^2^	*p*-value
		
	*n*	%	*n*	%	*n*		
Cerebral palsy	159	47.8	141	61.8	300	0.963	0.299
Cataract	67	20.1	44	19.2	111	4.360	0.0290*
Clubfoot	54	16.6	22	9.6	76	12.645	< 0.0100
Glaucoma	34	10.2	10	4.3	44	12.023	< 0.0100*
Cleft lip or palate	18	5.4	11	4.8	29	3.841	0.194

*Source*: Authors’ original data

Values of table given as (*χ*^2^ = 15.3; *df* = 4).

* *p* = 0.0040; statistically significant difference.

**FIGURE 1:**
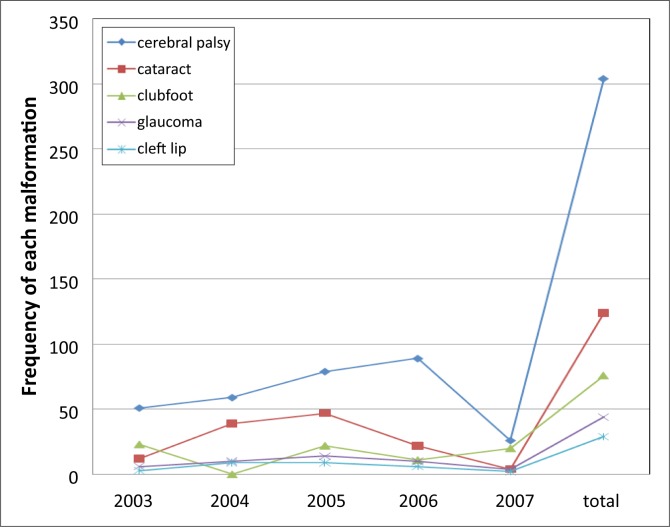
Trends of five common conditions relating to morbidity throughout the study period.

The trends of five different common malformations during the study period are most effectively comprehended by viewing Figure 1 which refers to cerebral palsy, cataract, clubfoot, glaucoma, and cleft lip. This figure shows high rates of cerebral palsy throughout the study. It was a mistake, and I am grateful to the reviewer.

## Discussion

### Outline of the results

Statistically significant differences were found for the distributions of the five aforementioned conditions regarding gender (*p* = 0.0040) and age (*p* = 0.0000) of participants.

Differences were observed between male participants and female participants regarding the incidence of glaucom (*p* < 0.0100), clubfoot (*p* = 0.0100), and cataract (*p* = 0.0290) for both male and female participants (Table 2). The incidence of cerebral palsy (*p* = 0.0000), of clubfoot (*p* = 0.0000), and of glaucoma (*p* = 0.0010) were statistically different between the two age ranges.

**TABLE 2b:** Variance of different morbidities by age.

Age ranges	≤ 5 years (*n* = 485)		6–18 years (*n* = 75)		Total	*χ*^2^	*p*-value
		
	*n*	%	*n*	%	*n*		
Cerebral palsy	291	60	9	12	300	263.203	< 0.0001*
Cataract	68	14	43	57.3	111	2.860	0.0176*
Clubfoot	72	14.8	4	5.3	76	59.066	< 0.0001*
Glaucoma	37	7.6	7	9.3	44	11.672	0.0001*
Cleft lip or palate	17	3.5	12	16	29	0.427	0.514

*Source*: Authors’ original data

Values of table given as (*χ*^2^ = 114; *df* = 4).

* *p* = 0; statistically significant difference.

Classically, it has been established that clubfoot affects twice as many boys as girls.^17^ Cataracts, clubfoot, and cerebral palsy were observed at consistently high rates during the years covered by the study, compared to other deformities (Figure 1). There are several reasons for the high incidence of these conditions amongst participants. Firstly, Christopher Blind Mission, the main partner in the field for several years in the region and from which the model of rehabilitation has been taken, has a special interest in eye and orthopaedics conditions. The same reason would explain the lack of medical conditions in our morbidity profile. Secondly, the higher visibility of these surgical conditions for diagnostic purposes and the social stigmatisation attached to their presence could play a role in the preferential surgical healthcare-seeking patterns of patients and could explain this disproportion of conditions. Clubfoot has implications at both the individual as well as asocial level. Neglected or inadequately corrected clubfoot can cause physical impairment that results in difficulty to move around and to walk and an inability to perform basic tasks pertaining to the local needs, such as carrying water, collecting food, and attending school.^18^ Thirdly, the fact that the composition of the rehabilitation team was biased towards surgery could also explain the trends. This third reason, in part, could explain the lack of medical conditions such as congenital heart diseases, complications of sickle cell anaemia, epilepsy, and so forth, in our sample.

We are most concerned by the high rate of cerebral palsy in our study (81.5%). Cerebral palsy is either isolated or associated with other deformities in complex syndromes such as developmental dysplasia of the hip and neuromuscular disorders including *spina bifida*, *arthrogryposis*, and *myelodysplasia*.^17^ In our case, there were no apparent associated conditions. When isolated, cerebral palsy can arise from obstetrical complications following asphyxia at birth, low birth weight, or in most cases from unknown causes. Three conditions (cerebral palsy, clubfoot, and glaucoma) are significantly predominant amongst children less than 5 years old. This is probably because of high early mortality rates linked to conditions such as cerebral palsy and also the social role associated with clubfoot, which plays a role in early care-seeking behaviours.

The mixed morbidity pattern observed in this study is more complicated. Some minor deformities such as polydactyly and syndactyly have been reported without leading to either disability or chronic conditions. Those minor conditions may just translate to either late care-seeking behaviour or misinformation of parents regarding rehabilitation centres in Africa. In addition, the frequent home deliveries in our settings is denying new-born babies access to a full neonatal assessment after birth by trained personnel during which such small deformities could be detected, corrected, or referred promptly for appropriate management.

Patients with other severe complications such as an imperforated anus are seeking rehabilitation in an ordinary manner. Unlike those seeking consultation for minor ailments as described earlier, this second group underscores the gravity of imperforated anus. It appears that in addition to the high level of ignorance, to complain about a condition is a socially constructed concept that can vary with several parameters, including culture. If a condition is well accepted in a setting, it can prevent consultation with a professional or result in late consultation, with consequent poor or limited results, as illustrated by neglected conditions such as *spina bifida*. This mixture goes beyond the condition's type encountered and includes conditions in need of curative measures together with those in need of pure rehabilitation. It translates probably either the label given to health providers as primarily ‘curative guys’ by the community or the misinformation of the population about the potential and scope of rehabilitative activities. More cases brought to attention were congenital in nature, and perhaps their high proportion could explain the possible association made previously in the neighbouring region of Ituri regarding possible correlations between the high occurrences of congenital malformations with the on-going armed conflicts.^19^

This study is included in the large spectrum of evidence published by various scholars. Globally, in the literature, community-based rehabilitation is not a scarce concept per se, as it has been widely used in different countries and for different purposes. Many conditions within it have been addressed such as rehabilitation in spinal injury,^2, 3^ in elderly integration,^20^ quality of life,^21, 22^ cerebral palsy,^23^ mental health,^6, 24, 25^ leprosy,^26^ and stroke.^27^ Other studies have examined the attitude of the community toward people with disabilities.^28^ A study focused exclusively on describing conditions observed in a rehabilitation unit was not identified as such to our knowledge; many studies dealt with either one condition, the setup of the rehabilitation, the process of CBR, or the outcomes of community based-rehabilitation programmes.

### Practical implications

This study is important because it provides a list of conditions with their distribution that are observed in the rehabilitation centre in Butembo. With this information, providers can now tailor their kits accordingly and their teams can be adjusted to meet the needs expressed by patients in a satisfactory manner. Programmes can also project expenses in terms of training, direct cost, and fund raising.

### Limitations

The trend of the conditions as a whole was not recorded for practical reasons because of the high number of conditions, most of them with small frequencies and/or being present for a year or two. We decided that the five most common conditions that were present during the study period could portray the trends effectively.

We could not generalise the results of the current study to the community because the data were from an outreach facility, although it was based on the results of clients seen in the community. That is to say, we cannot extrapolate the findings and apply them to the whole community of Butembo.

More light will be shed in this field by researching the workload and contribution of each member of the team in the management of different patients. Simple descriptive and/or case-control studies followed by analytical or experimental studies on cerebral palsy, the most common condition, would help to identify the magnitude of the problem, the risk factors, and probably the causes.

A community survey of the cases in need of rehabilitation could fill in this picture and reveal the real needs of the community served.

### Recommendations

In light of the results, we recommend the implementation of a triaging service to screen clients before they see the consulting team in order to minimise the waiting time of clients in need of urgent curative care. A multidisciplinary team could help to provide an entire package of comprehensive care and follow-up consultations after contact with the rehabilitation unit. This could also help to reach more people in need who are affected by medical conditions.

## Conclusion

The main conditions managed in CBR in Butembo, their trends throughout the years, and their gender distributions have been elucidated. There remain uncertainties with regard to the mandate of the rehabilitative team, which results in an assortment of cases presented to the team when they visit the centre. Further studies aimed at exploring some frequent conditions such as cerebral palsy would be of significance. Sensitisation of the community for early consultation and triage of clients in need of rehabilitation services are warranted.
